# Endurance exercise induces REDD1 expression and transiently decreases mTORC1 signaling in rat skeletal muscle

**DOI:** 10.14814/phy2.12254

**Published:** 2014-12-24

**Authors:** Miki Hayasaka, Haruka Tsunekawa, Mariko Yoshinaga, Taro Murakami

**Affiliations:** 1 Department of Nutrition, Shigakkan University, Yokone‐Machi, Ohbu, Japan

**Keywords:** REDD1, mTORC1, S6K1, 4E‐BP1, protein synthesis

## Abstract

Working muscle conserves adenosine triphosphate (ATP) for muscle contraction by attenuating protein synthesis through several different pathways. Regulated in development and DNA damage response 1 (REDD1) is one candidate protein that can itself attenuate muscle protein synthesis during muscle contraction. In this study, we investigated whether endurance exercise induces REDD1 expression in association with decreased mammalian target of rapamycin (mTOR) complex I (mTORC1) signaling and global protein synthesis in rat skeletal muscle. After overnight fasting, rats ran on a treadmill at a speed of 28 m/min for 60 min, and were killed before and immediately, 1, 3, 6, 12, and 24 h after exercise. REDD1 mRNA and corresponding protein levels increased rapidly immediately after exercise, and gradually decreased back to the basal level over a period of 6 h in the gastrocnemius muscle. Phosphorylation of mTOR Ser2448 and S6K1 Thr389 increased with the exercise, but diminished in 1–3 h into the recovery period after cessation of exercise. The rate of protein synthesis, as determined by the surface sensing of translation (SUnSET) method, was not altered by exercise in fasted muscle. These results suggest that REDD1 attenuates exercise‐induced mTORC1 signaling. This may be one mechanism responsible for blunting muscle protein synthesis during exercise and in the early postexercise recovery period.

## Abstract (precis)

We show that REDD1 expression is rapidly induced by an acute bout of endurance exercise in association with a decrease in mTORC1 signaling in rat muscle. The rate of mixed‐muscle protein synthesis was, however, not altered by exercise in fasted state. These results suggest that REDD1‐induced suppression of mTORC1 signaling may be one mechanism to blunt muscle protein synthesis during exercise and postexercise early recovery period.

## Introduction

In many organisms, a hierarchy of adenosine triphosphate (ATP)‐consuming processes is strictly but flexibly controlled to maintain energy homeostasis (Buttgereit and Brand 1995). During continuous muscle contraction and relaxation, during exercise, for example, ATP expenditure for synthesizing muscle proteins is a lower priority than that required for muscular movement, wherein available ATP is utilized for rapid muscular movements necessary for animal survival (Bylund‐Fellenius et al. 1984). One of the mechanisms by which exercise presumably suppresses protein synthesis in working muscles is through downregulation of the mammalian target of rapamycin (mTOR) complex 1 (mTORC1, Gautsch et al. 1998), which regulates key cellular functions linked to the promotion of cell growth and metabolism (Kimball and Jefferson 2010; Laplante and Sabatini 2012; Shimobayashi and Hall 2014). Adenosine monophosphate (AMP)‐activated protein kinase (AMPK, Williamson et al. 2006; Dreyer et al. 2006) and REDD1 (Murakami et al. 2011) are two candidates proposed to drive exercise‐induced downregulation of mTORC1 signaling. Furthermore, Ca^2+^‐dependent inactivation of eukaryotic elongation factor 2 (eEF2), which regulates the elongation step in protein synthesis, has also been reported to be involved in suppressing protein synthesis in working muscles (Rose et al. 2005, 2009).

*Redd1* was originally identified as a gene that is transcriptionally upregulated in response to a variety of conditions, inducing cellular stressors like hypoxia (Shoshani et al. 2002). Subsequently, REDD1 was identified as a potent inhibitor of mTORC1 (Brugarolas et al. 2004). REDD1 mediates this inhibition by activating TSC1/TSC2 (Kimball et al. 2008). Because muscle protein synthesis is induced during the postexercise recovery period (Biolo et al. 1995), it is conceivable that REDD1, with a short half‐life of less than 5 min (Kimball et al. 2008), could be a transient inhibitor of mTORC1 during exercise. Indeed, we have demonstrated that an acute bout of endurance exercise, repeated bouts of which lead to mitochondrial biogenesis thereby improving muscular endurance, rapidly induces the expression of REDD1 mRNA and protein, and downregulates mTORC1 signaling in rat skeletal muscles (Murakami et al. 2011). However, the relationship between REDD1 expression, mTORC1 signaling, and the rate of global protein synthesis in the muscles during the postexercise period remains to be elucidated.

In contrast to endurance exercise, it has been shown that a single bout of resistance exercise, repeated bouts of which leads to muscular hypertrophy thereby the increase in muscle strength and power, represses the expression of REDD1 mRNA in human muscle (Drummond et al. 2008; Liu et al. 2010). Recently, Gordon et al. (2014) have reported that electrically induced muscle contraction, repeated bouts of which lead to muscle hypertrophy (Baar and Esser 1999), also represses REDD1 expression in association with activated mTORC1 signaling and muscle protein synthesis. The apparent difference between the REDD1 response to endurance (Murakami et al. 2011) and resistance exercise (Drummond et al. 2008; Liu et al. 2010; Gordon et al. 2014) suggests that REDD1 could be involved in specific adaptive responses of the muscle to endurance or resistance training.

In this study, our primary goal was to identify the time point at which exercise‐induced REDD1 expression returns to the basal level during the recovery period following an acute bout of endurance exercise. The second goal was to investigate whether changes in REDD1 expression alter the rate of muscle protein synthesis due to decreased/increased mTORC1 signaling.

## Materials and Methods

**Figure 1.**
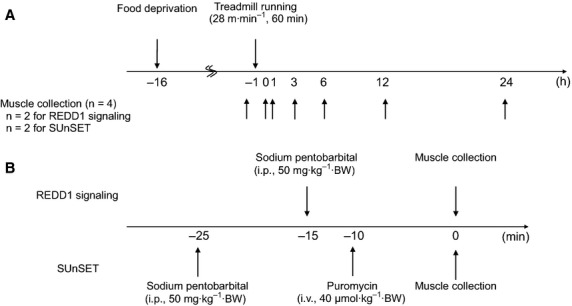
Experimental protocol. To maintain consistency, the animal procedure shown in panel A was repeated twice in an identical manner (*n* = 28 each time). (A) On the day of the experiment, after 16 h of fasting, rats were divided into pre‐exercise (*n* = 4) and exercise (*n* = 24) groups. The rats in the exercise group ran on the treadmill at 28 m/min for 60 min, and were killed immediately, 1, 3, 6, 12, or 24 h after exercise (*n* = 4 for each time point). (B) At each time point, the rats were anesthetized with sodium pentobarbital (i.p., 50 mg/kg of body weight) and the gastrocnemius muscle was quickly dissected out 15 min later. To determine the rate of mixed‐muscle protein synthesis, 50% of the rats at each time point (*n *= 2) were administered puromycin (i.v., 40 *μ*mol/kg of the body weight) 10 min after anesthesia and killed exactly 10 min later. The rats in the pre‐exercise group were treated in the same manner, except that they did not run on the treadmill on the day of the experiment.

Fifty‐six male Sprague–Dawley rats (5 weeks old) were obtained from Japan SLC Inc. (Hamamatsu, Japan). The Experimental Animal Care Committee of Shigakkan University approved all procedures involving animals. Rats were housed in pairs at 22°C and 50% humidity, with a 12:12‐h light–dark (12L/12D) photoperiod. Food (CE‐2; CLEA Japan, Tokyo, Japan) and water were provided ad libitum. To maintain consistency, the animal procedures described below were repeated twice in an identical manner (*n* = 28 each time). Rats were acclimated to a motor‐driven treadmill designed specifically for rats (Natsume Seisakusho, Tokyo, Japan). For a period of 14 days before the experiment, the treadmill was operated at a speed of 10–28 m/min for 20–60 min/day, (5 times/week, total 10 times). On the day of the experiment, after 16 h of fasting, rats were divided into pre‐exercise (*n* = 4) and exercise (*n* = 24) groups. Rats of the exercise group ran on the treadmill at 28 m/min for 60 min, and were killed immediately, 1, 3, 6, 12, and 24 h after exercise (*n* = 4 for each time point, Fig. 1A). At each time point after exercise, the rats were anesthetized with sodium pentobarbital (50 mg/kg of body weight). The gastrocnemius muscle was quickly removed and freeze‐clamped with an aluminum block that had been precooled in liquid nitrogen (Fig. 1B). To determine the rate of mixed‐muscle protein synthesis, 50% of the rats at each time point (*n* = 2) were administered puromycin (Sigma) intravenously (40 *μ*mol/kg of body weight) after 10 min of anesthesia and killed exactly 10 min later. After collecting the muscle samples, the rats were killed by severing the diaphragm and the heart. The rats in the pre‐exercise group were treated similarly, except that they did not run on the treadmill on the day of the experiment.

Phosphorylation of mTOR Ser2448, ribosomal protein S6 kinase, 70 kDa, polypeptide 1 (S6K1) Thr389, 4E‐BP1, AMPK Thr172, and extracellular signal‐regulated kinase 1/2 (ERK 1/2) T202/Y204, as well as expression of the REDD1 protein were all measured by western blot as described previously (Murakami et al. 2011). Antibodies for detecting mTOR, S6K1, 4E‐BP1, AMPK, and ERK 1/2 were obtained from Cell Signaling Technology (Danvers, MA). The antibody for detecting REDD1 was obtained from Proteintech Group (Chicago, IL). To detect total protein levels of mTOR, S6K1, and AMPK, after detecting the phosphorylated forms of the proteins, blots were stripped of antibody and reprobed with antibodies that recognize each protein, independent of the phosphorylation state. In the case of ERK 1/2, stripping of the antibody was unsuccessful for an unknown reason; therefore, membranes were prepared in duplicate to measure phosphorylated and total ERK 1/2. Membranes were stained with Ponceau‐S to ensure effective transfer and equal protein loading for all western blot analyses.

Total RNA was extracted with Isogen II (Nippon Gene, Tokyo, Japan), and complementary DNA was generated using the PrimeScript RT reagent kit (Takara Bio, Ohtsu, Japan). REDD1 mRNA expression was measured using quantitative reverse transcription polymerase chain reaction (qRT‐PCR) as described previously (Murakami et al. 2011).

The rate of protein synthesis was measured by the SUnSET method (Schmidt et al. 2009) using an antipuromycin monoclonal antibody (KeraFAST, Boston, MA).

To evaluate the differences among the various groups, data were analyzed using a one‐way factorial analysis of the variance. A Fisher's protected least significant difference (PLSD) test was used for the post hoc test when a significant difference was observed. Values of *P *< 0.05 were defined as statistically significant.

## Results

### REDD1 mRNA and protein expression in the gastrocnemius muscle during the postexercise recovery period after endurance exercise

**Figure 2.**
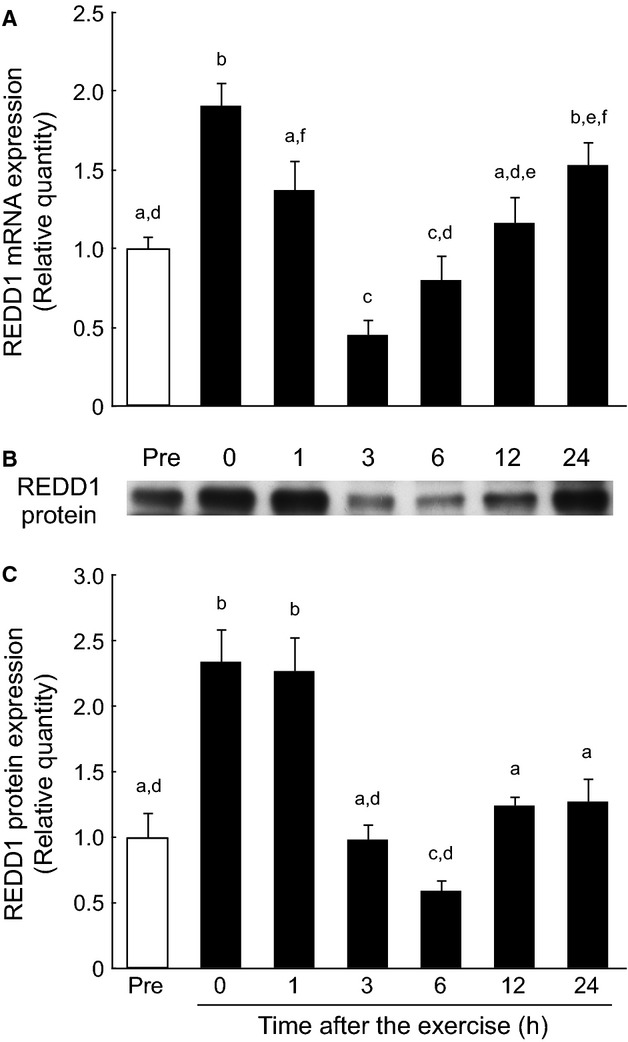
Induction of REDD1 expression in the rat gastrocnemius muscle after a bout of endurance exercise. REDD1 expression rapidly increased with exercise and gradually decreased to the basal level after 24 h. (A) REDD1 mRNA expression. The relative amount of REDD1 mRNA was normalized to that of the 18S rRNA and expressed relative to the pre‐exercise control. (B) Representative immunoblot of REDD1. (C) REDD1 protein expression. Values are expressed as mean ± SE for four rats. Means not sharing the same superscript are significantly different (*P* < 0.05).

Expression of REDD1 mRNA significantly increased with exercise, but returned to the pre‐exercise level 1 h after cessation of exercise (Fig. 2A). Thereafter, mRNA expression further decreased below the basal level 3 h after cessation of exercise, and increased to the pre‐exercise level 6 h after cessation of exercise. REDD1 protein levels also increased rapidly with exercise, and continued to increase at least 1 h into the postexercise recovery period (Fig. 2B and C). REDD1 protein expression returned to the pre‐exercise level 3 h after cessation of exercise.

### Phosphorylation of mTOR, S6K1, and 4E‐BP1 in the gastrocnemius muscle during the postexercise recovery period after endurance exercise

**Figure 3.**
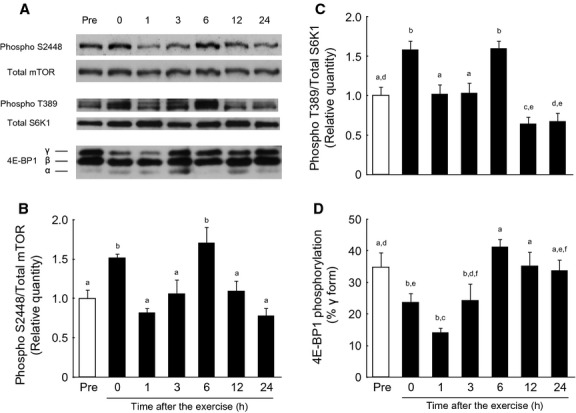
Changes in phosphorylation of mTOR Ser2448, S6K1 Thr389, and 4E‐BP1 in the rat gastrocnemius muscle after a bout of endurance exercise. (A) Representative immunoblots of mTOR Ser2448, S6K1 Thr389, and 4E‐BP1. (B–D) Phosphorylation of mTORC1 signaling during the postexercise recovery period. The relative levels of mTOR2448 and S6K1 Thr389 phosphorylation were normalized to that of the total mTOR and S6K1 proteins, respectively, and expressed relative to the pre‐exercise control. Values are expressed as mean ± SE for four rats. Means not sharing the same superscript are significantly different (*P* < 0.05).

Phosphorylation of mTOR Ser2448 increased transiently and significantly with exercise in the gastrocnemius muscle (Fig. 3A and B). The increase in mTOR Ser2448 phosphorylation returned to the pre‐exercise level 1 h after cessation of exercise. After 1 h post exercise, phosphorylation of mTOR Ser2448 increased gradually and significantly above the basal level through 6 h after cessation of exercise. Phosphorylation of mTOR Ser2448 returned to the pre‐exercise level 12 h after cessation of exercise, and remained constant for another 12 h.

Likewise, the phosphorylation of S6K1 Thr389 also increased transiently and significantly with exercise in the gastrocnemius muscle (Fig. 3A and C). The increase in the phosphorylation of S6K1 Thr389 returned to the pre‐exercise level 1 h after cessation of exercise. Phosphorylation of S6K1 subsequently increased significantly through 6 h after the cessation of the exercise. Thereafter, phosphorylation of S6K1 Thr389 decreased and returned to the pre‐exercise level.

Although both S6K1 and 4E‐BP1 are well‐known downstream targets of mTORC1 (Kimball and Jefferson 2010; Laplante and Sabatini 2012; Shimobayashi and Hall 2014), the timing of exercise‐induced phosphorylation of 4E‐BP1 differed from that of S6K1 Thr389 (Fig. 3A and D). 4E‐BP1 phosphorylation significantly decreased with exercise, and continued to decrease further at least 1 h into the postexercise recovery period. The exercise‐induced decrease in phosphorylation of 4E‐BP1 returned to the pre‐exercise level 3 h after cessation of exercise. Following 6 h after cessation of exercise, phosphorylation of 4E‐BP1 remained constant for another 18 h.

### Phosphorylation of AMPK in the gastrocnemius muscle during the postexercise recovery period after endurance exercise

**Figure 4.**
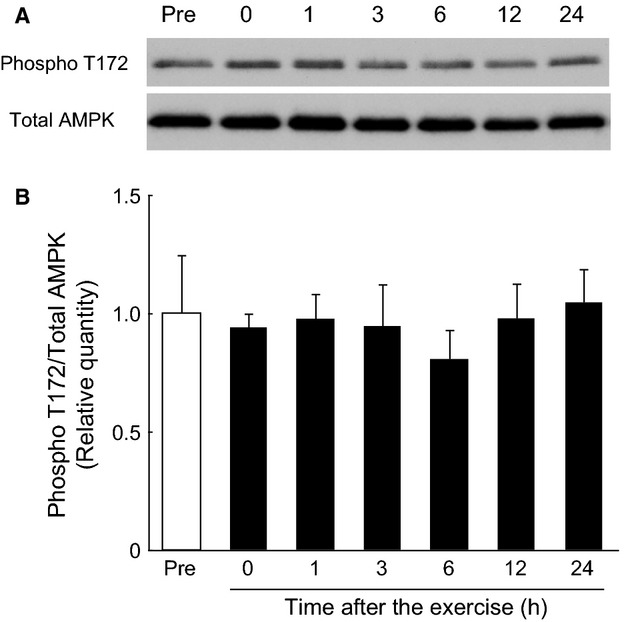
No change in the phosphorylation of AMPK Thr172 in the rat gastrocnemius muscle after a bout of endurance exercise. Phosphorylation of AMPK Thr172 was not altered during the 24‐h recovery period after endurance exercise. (A) Representative immunoblot. (B) Phosphorylation of AMPK Thr172 during the postexercise recovery period. The relative level of phosphorylation of AMPK Thr172 was normalized to that of total AMPK protein and expressed relative to the pre‐exercise control. Values are expressed as mean ± SE for four rats.

Phosphorylation of AMPK Thr172 was unchanged in the gastrocnemius muscle throughout the 24‐h postexercise recovery period after endurance exercise (Fig. 4).

### Phosphorylation of ERK 1/2 in the gastrocnemius muscle during the postexercise recovery period after endurance exercise

**Figure 5.**
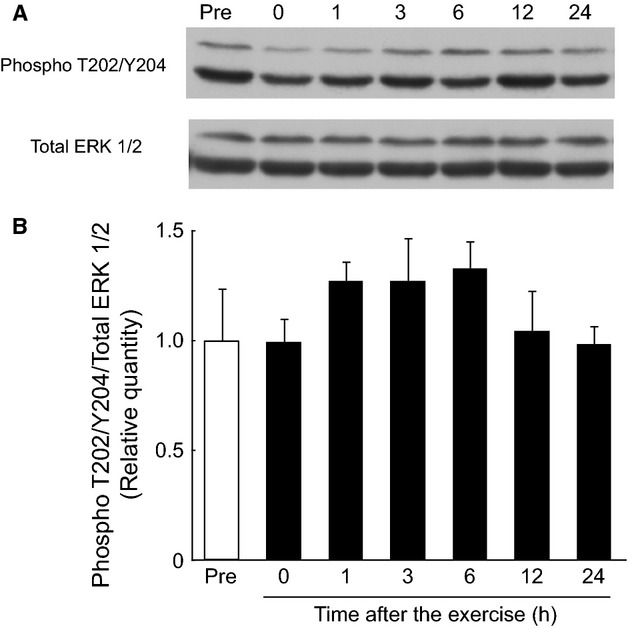
No change in the phosphorylation of ERK 1/2 Thr202/Tyr204 in the rat gastrocnemius muscle after a bout of endurance exercise. Phosphorylation of ERK 1/2 Thr202/Tyr204 was not altered during the 24‐h recovery period after endurance exercise. (A) Representative immunoblot. (B) Phosphorylation of ERK 1/2 Thr202/Tyr204 during the postexercise recovery period. The relative level of phosphorylation of ERK 1/2 Thr202/Tyr204 was normalized to that of the total ERK protein and expressed relative to the pre‐exercise control. Values are expressed as mean ± SE for four rats.

Phosphorylation of ERK 1/2 Thr202/Tyr204 was also unchanged in the gastrocnemius muscle throughout the 24‐h postexercise recovery period after endurance exercise (Fig. 5).

### Mixed‐muscle protein synthesis in the gastrocnemius muscle during the postexercise recovery period after endurance exercise

**Figure 6.**
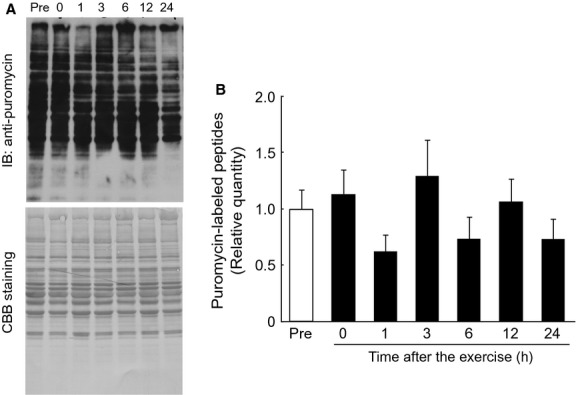
No change in the rate of mixed‐muscle protein synthesis after a bout of endurance exercise. The rate of mixed‐muscle protein synthesis in the fasted gastrocnemius muscle, measured as the rate of incorporation of puromycin into newly synthesized polypeptides (by the SUnSET method). Rate of protein synthesis was not altered during the 24‐h recovery period after endurance exercise. (A) Representative immunoblot. (B) The rate of mixed‐muscle protein synthesis. Values are expressed as mean ± SE for four rats.

To investigate whether induction of REDD1 expression and corresponding alterations in mTORC1 signaling could lead to changes in the rate of protein synthesis, the rate of mixed‐muscle protein synthesis in the gastrocnemius muscle was determined during the postexercise recovery period after endurance exercise. In contrast to our original hypothesis, the rate of mixed‐muscle protein synthesis in the muscle was not altered throughout the 24‐h postexercise recovery period after exercise (Fig. 6).

## Discussion

In this study, we demonstrate that REDD1 expression is rapidly induced after an acute bout of endurance exercise. This expression gradually returned to the basal level 3 h after the cessation of exercise. Phosphorylation of mTOR Ser2448 and S6K1 Thr389 increased transiently with exercise, but diminished at least 3 h into the recovery period after cessation of exercise. Phosphorylation of 4E‐BP1 decreased with exercise, and continued to decrease further until at least 1 h after cessation of exercise. The exercise‐induced decrease in phosphorylation of 4E‐BP1 returned to the pre‐exercise level 3 h after cessation of exercise. The rate of mixed‐muscle protein synthesis, as determined by the SUnSET method, was not altered by exercise during the postexercise recovery period in the fasted muscle. These results suggest that REDD1 attenuates exercise‐induced mTORC1 signaling, and hence may be one mechanism responsible for blunting muscle protein synthesis during exercise and in the postexercise early recovery period.

A role for REDD1 in reducing mTORC1 signaling during exercise and during the postexercise early recovery period is supported by the reduction of 4E‐BP1 phosphorylation immediately after exercise and 1 h into postexercise recovery period. Presumably, this would lead to subsequent inhibition of translation initiation. However, mTOR Ser2448 phosphorylation increases immediately after cessation of exercise, corresponding with the time when REDD1 expression increases. It has been reported that phosphorylation of mTOR Ser2448 increases immediately after cessation of endurance exercise in both rodent (Edgett et al. 2013) and human (Mascher et al. 2007, 2011; Wilkinson et al. 2008; Camera et al. 2010) muscles. However, other studies have shown that phosphorylation of mTOR Ser2448 is not altered immediately after endurance exercise (Morrison et al. 2008) and low‐frequency electrical stimulation (Atherton et al. 2005) in rodent muscle. One upstream activator of mTORC1 during exercise is Akt (Drummond et al. 2009; Miyazaki and Esser 2009). Akt is activated by growth factors and/or muscle contraction and it can directly activate mTORC1 by phosphorylating mTOR Ser2448 or indirectly by phosphorylating and inactivating TSC2, an upstream repressor of mTORC1. Phosphorylation of Akt has been reported to either increase (Wilkinson et al. 2008; Wang et al. 2011; Edgett et al. 2013) or remain unchanged (Williamson et al. 2006; Mascher et al. 2007, 2011; Camera et al. 2010) during endurance exercise in rodent and human muscles. Thus, phosphorylation of Akt and mTOR Ser2448 is not always consistent with each other immediately following endurance exercise in muscle. Although the precise upstream mechanism increasing mTOR Ser2448 phosphorylation and subsequently activating mTORC1 is not defined, our present and the aforementioned observations suggest that factors regulating both increased (e.g., Akt) and decreased (e.g., AMPK and/or REDD1) mTOR Ser2448 phosphorylation may act on mTOR simultaneously. The balance of these factors, then, would define the activity state of mTOR during endurance exercise in the muscle. In this context, although increased REDD1 expression does not completely blunt phosphorylation of mTOR at Ser2448 in this study, attenuation of mTORC1 activity and subsequently in the rate of muscle protein synthesis may still occur during endurance exercise and in the early recovery period in muscle.

An obvious discrepancy in phosphorylation between two downstream targets of mTORC1, S6K1 and 4E‐BP1, was observed immediately after exercise in the muscle: when phosphorylation of mTOR Ser2448 increased, phosphorylation of S6K1 also increased, but phosphorylation of 4E‐BP1 decreased. It has been reported that phosphorylation of 4E‐BP1 decreases immediately after a bout of endurance (Williamson et al. 2006; Morrison et al. 2008; Camera et al. 2010) or resistance (Dreyer et al. 2006, 2008; Deldicque et al. 2008a,b) exercise in rodent and human muscles, even when phosphorylation of S6K1 is unchanged or increased. Although mTORC1 is known to phosphorylate and regulate both S6K1 and 4E‐BP1 directly, it has been shown that mTORC1 output to 4E‐BP1 is rapamycin‐insensitive whereas mTORC1 signaling to S6K1 is rapamycin‐sensitive (Wang et al. 2005). Recently, Kang et al. (2013) used in vitro analysis to demonstrate that mTORC1 kinase activity toward downstream phosphorylation sites varies widely. This is one mechanism through which mTORC1 effectors can respond differently to the same signals. Further studies are required to determine the discrepancy between the various effects of mTORC1 on downstream targets and its physiological role in energy and protein homeostasis during exercise in muscle.

AMPK may serve as a potent inhibitor of muscle mTORC1 during endurance exercise (Williamson et al. 2006). However, a decrease in protein synthesis as well as the suppression of mTORC1 signaling was observed in mouse muscle overexpressing kinase‐dead AMPK (Rose et al. 2009), thereby suggesting that proteins other than AMPK could be involved in suppressing mTORC1 signaling and muscle protein synthesis. In this study, the phosphorylation of AMPK Thr172 was not altered during 24‐h postexercise recovery period after the exercise in the muscle. Durante et al. (2002) showed that AMPK activity is highly suppressed after endurance training in red quadriceps muscle of rats, presumably due to reduced metabolic stress by adaptation of the muscle. This observation suggests that the rats used in this study would undergo relatively less metabolic stress from exercise on the experimental day due to prior acclimation to the treadmill running; consequently, phosphorylation of AMPK would likely not be altered by the exercise. In any event, our finding that endurance exercise induces REDD1 expression, even when AMPK phosphorylation is not altered, suggests that not only AMPK, but also REDD1, seems to inactivate protein synthesis during endurance exercise and in the postexercise early recovery period.

Despite apparent discrepancies between REDD1 expression and mTOR phosphorylation, and between S6K1 and 4E‐BP1 phosphorylation immediately after exercise, the timing of REDD1 expression and mTORC1 signaling were consistent with each other between 1 and 6 h into the postexercise recovery period. REDD1 expression gradually decreased and mTOR Ser2448, S6K1 Thr389, and 4E‐BP1 phosphorylation correspondingly increased. It has been reported that phosphorylation of Akt increases during the recovery period after endurance exercise in human muscle (Mascher et al. 2007, 2011; Wilkinson et al. 2008; Camera et al. 2010; Wang et al. 2011; Esbjörnsson et al. 2012). These observations suggest that the factors increasing phosphorylation of mTOR Ser2448 (e.g., Akt) would be more influential than those decreasing phosphorylation (e.g., REDD1). Hence, mTORC1 signaling and presumably subsequent muscle protein synthesis would recover during the postexercise early recovery period. MEK/ERK/RSK1 signaling has been suggested to increase with the phosphorylation of S6K1 Thr389 by muscle contraction, independent of mTORC1 (Liu et al. 2013). However, in this study, phosphorylation of ERK 1/2 Thr202/Tyr204 was not altered during the 24‐h postexercise recovery period. This result suggests that the increase in phosphorylation of S6K1 could be due to the activation of mTORC1 rather than MEK/ERK/RSK1 signaling.

The half‐life of REDD1 has been reported to be less than 5 min (Kimball et al. 2008). Because protein synthesis has been shown to increase after a bout of exercise (Biolo et al. 1995), REDD1 could be a transient, yet potent inhibitor of mTORC1 during exercise. In spite of the short half‐life of REDD1, the induction of REDD1 protein expression continued at least 1 h into the postexercise recovery period, suggesting that not only hypoxia (a potent inducer of REDD1 expression, Shoshani et al. 2002) but also other factors such as a low‐energy status (Sofer et al. 2005; McGhee et al. 2009) could be involved in the prolonged induction of REDD1 during the postexercise early recovery period.

A single bout of resistance exercise represses expression of REDD1 mRNA in human muscle (Drummond et al. 2008; Liu et al. 2010). Recently, Gordon et al. (2014) reported that electrically induced muscle contraction, repeated bouts of which lead to muscular hypertrophy (Baar and Esser 1999), also represses expression of REDD1 mRNA and protein in association with the activation of mTORC1 signaling and muscle protein synthesis. It has also been reported that immobilization of rat hindlimb induces expression of REDD1 mRNA in association with the suppression of mTORC1 signaling and muscle protein synthesis (Kelleher et al. 2013, 2014). Variable REDD1 responses to endurance exercise (Murakami et al. 2011), resistance exercise (Drummond et al. 2008; Liu et al. 2010), muscle contraction (Gordon et al. 2014), and/or muscle immobilization (Kelleher et al. 2013, 2014) emphasize the complexity of REDD1 in regulating muscle protein synthesis through blunting mTORC1. The precise mechanisms underlying REDD1‐mediated regulation of muscle protein synthesis in response to divergent upstream stimuli remain to be elucidated.

It should be noted that, in this study, we used relatively young rats (7‐week old at sacrifice), because older, heavy rats are sometimes difficult to run on a treadmill. The rate of protein synthesis declines during growth and development in mammals (Waterlow and Stephen 1967). In addition, this parameter parallels the reduction in the intensity of energy metabolism that occurs during the growth period (Kleiber 1961). These observations suggest that requirement of ATP during exercise might be higher in younger than in older mammals. Hence, induction of REDD1 expression would be more obvious in younger, growing mammals than mature, adult mammals. It remains to be determined whether REDD1 expression is also induced in older adult mammals.

In contrast to our original hypothesis, mixed‐muscle protein synthesis was neither suppressed by exercise nor recovered to the basal level during postexercise recovery period. One reason why protein synthesis was not altered during exercise or in the postexercise recovery period could be fasting. In this study, rats fasted for 16 h prior to endurance exercise on the experimental day to exclude the effects of diet on mTORC1 signaling. It has been reported that, in the fasting state, muscle protein synthesis is increased (Carraro et al. 1990; Sheffield‐Moore et al. 2004; Mascher et al. 2011), not altered (Tipton et al. 1996), or decreased (Gautsch et al. 1998) during the postexercise recovery period after endurance exercise in human and rodent. These observations suggest that many factors, including species, muscle type, exercise intensity, time at which measured, duration of fasting, and the level of training could be involved in the regulation of muscle protein synthesis following endurance exercise. Because muscle protein synthesis could be modulated by extracellular amino acid availability (Bohé et al. 2003), it is possible that in the fed state, where exogenous amino acids are supplied by the diet, a change in muscle protein synthesis would be observed during the postexercise recovery period with our experimental protocol.

A second possibility explaining why we did not observe changes in muscle protein synthesis could be related to the fraction of the muscle in which we measured the protein synthesis. Wilkinson et al. (2008) showed that endurance exercise increases muscle protein synthesis in the mitochondrial fraction, but not in the myofibrillar fraction during a 4‐h recovery period after exercise in nonfasting humans. They also showed that, in contrast to endurance exercise, resistance exercise increases both myofibrillar and mitochondrial protein synthesis. Furthermore, Koopman et al. (2011) reported that postexercise protein synthesis is only marginally higher in type I compared to type II muscle fibers following resistance exercise in humans. It is possible that mitochondrial protein synthesis and/or protein synthesis in type I fibers increases; an increase in protein synthesis in the mitochondrial fraction and/or type I fibers would be obscured by unchanged fractions and/or fibers. These changes in mixed‐muscle protein synthesis would be, then, undetectable by the experimental methods used in this study.

Finally, it is possible that, because the SUnSET method is less sensitive than isotope methods to assess differences in protein synthesis, we were simply unable to detect differences in the study. Although a growing number of studies in the literature are reporting changes in muscle protein synthesis by the SUnSET method (Goodman et al. 2011; Kelleher et al. 2013, 2014), the sensitivity of the SUnSET method is, in fact, lower than that of isotope methods. We applied the SUnSET method instead of isotope methods in this study owing to the limitation in our facilities. However, in a pilot study, we confirmed the validity of the SUnSET method in our experimental system by detecting an increase in mixed‐muscle protein synthesis following refeeding of rats after 48 h fasting (Fig. S1). Thus, we believe that the results obtained by the SUnSET method in this study accurately reflect protein synthesis in the muscle.

## Conclusion

In this study, we show that REDD1 expression is rapidly induced by an acute bout of endurance exercise. REDD1 expression returned to the basal level during the course of a 24‐h recovery period in fasted rat skeletal muscle. The induction of REDD1 correlated well with a decrease in mTORC1 signaling. The rate of protein synthesis was, however, not altered by exercise. These results suggest that REDD1‐induced suppression of mTORC1 signaling may be one mechanism to blunt protein synthesis in working muscles.

## Acknowledgments

We thank the members of the Laboratory of Nutrition of Chukyo Women's University and Shigakkan University for technical assistance.

## Conflict of Interest

The authors have no conflicts of interest to declare.
